# Strengthening policy research on infant and young child feeding: An imperative to support countries in scaling up impact on nutrition

**DOI:** 10.1186/s12889-017-4335-3

**Published:** 2017-06-13

**Authors:** Purnima Menon, Anne Marie Thow

**Affiliations:** 1 International Food Policy Research Institute, New Delhi, India; 2 Menzies Centre for Health Policy, School of Public Health, University of Sydney, Sydney, Australia

## Abstract

Enabling policy environments for nutrition require require evidence to support best practice and engagement with political and policy contexts, as well as leadership, resourcing, advocacy, and technical support. However, research on nutrition policy contexts is limited. The papers in this special supplement on policy contexts for infant and young child feeding (IYCF) in South Asia makes a valuable contribution to understanding the policy landscape and political dynamics in the region and the global literature. Studies included in this special supplement analyzed policy content and stakeholder influence on IYCF in Bangladesh, India, Nepal, Pakistan and Sri Lanka, and assess the role of advocacy in addressing multiple elements of the policy environment. These analyses highlight opportunities to harmonize and manage the demands and interests of multiple actors while strengthening policy to strategically support optimal IYCF as the ultimate goal. They also provide robust examples of research on policy environments and policy change. Further investments in research on policy contexts for nutrition can help to understand and support continued progress towards improved actions for nutrition.

In recent years, as efforts to bring nutrition and health on to leadership and policy agendas intensify globally, there is also emerging evidence of challenges in taking interventions to scale [1]. In nutrition, previous conceptual work has recognized the importance of moving from leadership to action [2] and the importance of aligning different elements of the policy landscape to support execution on the ground [3].

Even as gaps in coverage and reach of interventions are being identified and debated, it is also clear that the evidence identifying upstream factors that support effective implementation of a comprehensive approach to nutrition policy is growing too slowly. Much remains to be learnt. Previous work on policy issues in nutrition has focused on getting nutrition on the policy agenda, on leadership and capacity and on policy coordination [4, 5]. More recently, a set of studies on change processes in nutrition in multiple countries [6] has highlighted how diverse factors in the policy landscape affect the implementation of interventions. These factors include leadership, commitment to nutrition, financial support, alliance-building to harmonize advocacy asks and policy directions, technical and systems support for intervention delivery, and investment in creating and supporting frontline platforms for delivery. … Further research investigating these topics in nutrition, especially studying policy environment and implementation needs together and cohesively is, thus, crucial to strengthening country-specific evidence-building and identifying more generalizable cross-cutting knowledge on actions that facilitate translation of policy to action on the ground.

In South Asia, the burden of malnutrition is high and poor IYCF practices contribute significantly to both undernutrition and the emerging issues of overweight and obesity. However, research on nutrition policy landscapes and processes is limited in South Asia, despite acknowledgement that politics, policy processes and implementation challenges are key factors in shaping the nutrition agenda in specific countries in the region [7, 8].

This special supplement, makes a valuable contribution in bringing together a set of papers that systematically examine the factors that support IYCF policy implementation.Explicit analysis of policy-related issues can help identify issues that both the creation of supportive policy environments and the delivery of effective interventions The first five papers in this supplement present the findings from studies conducted in five countries by researchers from the South Asian Infant Feeding Research Network (SAIFRN) [9–13]. These studies examined policy content and stakeholder influence on IYCF in Bangladesh, India, Nepal, Pakistan and Sri Lanka.

At a regional level, the findings highlight a high level of political will for improving child nutrition; the integration of IYCF into a wide range of policy agendas throughout health and social welfare; and strong policies supporting provision of accurate information to mothers regarding IYCF and protection from commercial interests through the Code of Marketing of Breastmilk Substitutes [14]. Social network analysis across all studies highlights the complex network of local, national, regional and global stakeholders influencing IYCF policy in the region. This analysis identified around 40 influential stakeholders in Nepal, 70 in Bangladesh and 100 in Pakistan, India and Sri Lanka. These stakeholders included actors and institutions from government (both health and non-health agencies), development partners, the academic sector and local NGOs. At a regional level it was evident that United Nations Children’s Emergency Fund (UNICEF) and the World Health Organization (WHO) play a significant and influential role in supporting IYCF policy [15].

The papers also indicate significant strengths in IYCF policy at the country level. In all countries, specific political commitment to improving young child nutrition was evident in national, whole of government policy documents, such as National Development Plans. In India and Sri Lanka, this was supported by Nutrition Councils, which were highly influential, with the intent to gather a diverse range of stakeholders.

However, significant opportunities for strengthening IYCF policy in South Asia remain. Multisectoral approaches to improving IYCF requires strategic engagement of relevant actors and continued high level political will. Translation of policy objectives into implementation-level documents remains a challenge, particularly for field level staff training and operationalizing maternity leave commitments. The reliance on influential donors raises questions regarding sustainability and national ownership, particularly in Pakistan. Finally, monitoring and evaluation needs to be strengthened in all countries.

From a methodological perspective, this set of papers also highlight that an analysis of the policy environments for IYCF using a common perspective, but a country-specific lens, is feasible for national-level public health researchers to undertake with minimal support. This is important because these areas of research are quite unusual for the nutrition community; research on policy issues in many countries is often undertaken by those unaccustomed to studying specific fields of study. Although bringing technical expertise to the study of policy actors and policy processes is unusual, we anticipate that it will help to create more politically aware technical researchers, and support better research-to-policy relationships and linkages over time.

The final paper in this series speaks to the possible role of targeted issue-focused advocacy that addresses multiple elements of the policy environment [16]. This piece of work is unusual in that it examines shifts in national policy networks, policy discourse and the written policy environment for IYCF in three different countries, all targeted by single global advocacy initiative which developed and implemented tailored country advocacy strategies. In doing so, it emphasizes that it is indeed possible for thoughtful, technically solid, alliance-focused and well-resourced advocacy organizations to take on an issue that is not often politically chosen – that of nutrition and infant feeding – and raise awareness of it on the policy agenda.

Reflecting on the multiple papers on this series, we identify a set of key actions that can help strengthen IYCF policy environments in South Asia, maintain the profile of IYCF on policy and political agendas, *and* ensure supportive environments are established and maintained.

First, it is absolutely crucial to recognize the diversity and complexity of the actor environment in the IYCF policy space. Government, technical agencies, funders, civil society, the media and the industry are all involved. These actors may be supportive to the issue of IYCF (at best), and unsupportive, or even antagonistic and confrontational (at worst). Movement on the policy agenda must, therefore, examine how best to harmonize and manage the demands and interests of multiple actors while keeping support to optimal IYCF as the ultimate goal.

Second, researchers, must recognize the need for multiple elements to coalesce to support implementation of effective interventions. Policy and high-level support or commitment are only a part of the solution. Without financing, effective and large-scale delivery platforms, and adequate capacity building, policy translation will be limited. Implementation issues, including quality and retention of human resources, supervisory structure and funding, are critical to the successful translation of policy into action.

Third, the nutrition community must critically examine role of the formula industry in undermining effective policy implementation. Mothers, especially those who work outside the home, face real hurdles in attempting to ensure their babies are fed. The changing needs for IYCF support in rapidly changing contexts in South Asia means that the policy environment must include maternity leave without loss of pay, or other maternity protection and leave mandates.

Fourth, that it is essential to continue to invest in research that examines policy issues in nutrition, and researchers should return to these contexts to examine progress and changes over time. Careful policy analysis can help to ensure that recommendations for action on IYCF – and particularly, for scaling up effective interventions – are grounded in a solid understanding of the existing policy environment. For example, the analysis of strengths and weakness in policy content presented here, together with in-depth analysis of stakeholder influence, can be used to develop clear and specific recommendations for policy change and to develop effective advocacy strategies.

In closing, we reiterate the importance of the policy environment, the actors therein, and the changes in these environments over time for driving effective implementation. We call on both global and national researchers with an interest in public health outcomes and scaling up interventions to continue to invest in policy process research alongside more traditional research on testing and implementing interventions to improve infant and young child feeding.
